# Log odds of positive lymph nodes as a novel prognostic predictor for colorectal cancer: a systematic review and meta-analysis

**DOI:** 10.1186/s12885-022-09390-x

**Published:** 2022-03-18

**Authors:** Yiding Li, Guiling Wu, Yujie Zhang, Ben Han, Wanli Yang, Xiaoqian Wang, Lili Duan, Liaoran Niu, Junfeng Chen, Wei Zhou, Jinqiang Liu, Daiming Fan, Liu Hong

**Affiliations:** 1 State key Laboratory of Cancer Biology and National Clinical Research Center for Digestive Diseases, Xijing Hospital of Digestive Diseases, Fourth Military Medical University, 127 Changle West Road, Xi’an, Shaanxi Province 710032 P.R. China; 2 School of Aerospace Medicine, Fourth Military Medical University, Xi’an, 710032 China; 3 Department of Histology and Embryology, School of Basic Medicine, Xi’an Medical University, Xi’an, 710021 China; 4 Department of Nutrition, Xinqiao Hospital, Army Military Medical University, Chongqing, 40038 China

**Keywords:** The log odds of positive lymph nodes, Colorectal cancer, Prognosis

## Abstract

**Background:**

Colorectal cancer (CRC) is the third most prevalent cancer in the world, which remains one of the leading causes of cancer-related deaths. Accurate prognosis prediction of CRC is pivotal to reduce the mortality and disease burden. Lymph node (LN) metastasis is one of the most commonly used criteria to predict prognosis in CRC patients. However, inaccurate surgical dissection and pathological evaluation may lead to inaccurate nodal staging, affecting the effectiveness of pathological N (pN) classification in survival prediction among patients with CRC. In this meta-analysis, we aimed to estimate the prognostic value of the log odds of positive lymph nodes (LODDS) in patients with CRC.

**Methods:**

PubMed, Medline, Embase, Web of Science and the Cochrane Library were systematically searched for relevant studies from inception to July 3, 2021.

Statistical analyses were performed on Stata statistical software Version 16.0 software. To statistically assess the prognostic effects of LODDS, we extracted the hazard ratio (HR) and 95% confidence interval (CI) of overall survival (OS) and disease-free survival (DFS) from the included studies.

**Results:**

Ten eligible articles published in English involving 3523 cases were analyzed in this study. The results showed that LODDS1 and LODDS2 in CRC patients was correlated with poor OS compared with LODDS0 (LODDS1 vs. LODDS0: HR = 1.77, 95% CI (1.38, 2.28); LODDS2 vs. LODDS0: HR = 3.49, 95% CI (2.88, 4.23)). Meanwhile, LODDS1 and LODDS2 in CRC patients was correlated with poor DFS compared with LODDS0 (LODDS1 vs. LODDS0: HR = 1.82, 95% CI (1.23, 2.68); LODDS2 vs. LODDS0: HR =3.30, 95% CI (1.74, 6.27)).

**Conclusions:**

The results demonstrated that the LODDS stage was associated with prognosis of CRC patients and could accurately predict the prognosis of patients with CRC.

**Supplementary Information:**

The online version contains supplementary material available at 10.1186/s12885-022-09390-x.

## Introduction

Colorectal cancer (CRC) is one of the most common malignant tumors in the world, with high morbidity and mortality. It is estimated that there were over 1.8 million new cases in 2018, and at the same time, more than 881,000 deaths were estimated to have occurred [1]. Lymph node (LN) metastasis in patients with CRC is considered a reliable predictor of prognosis and a determinant for therapeutic decision-making [2, 3]. Currently, the most authorized tool for CRC staging assessment is the American Joint Committee on Cancer/International Union Against Cancer Classification (AJCC/UICC) tumor node metastasis (TNM) system, which classifies the pathological N (pN) stages according to the number of metastatic lymph nodes [4]. For optimal staging of CRC, the analysis of 12 or more lymph nodes is necessary for CRC patients, which was proposed by the AJCC/UICC. Due to inaccurate surgical dissection and pathological evaluation, an inadequate number of nodes examined may result in under-staging and improper treatment, known as “stage migration” [5–7]. Thus, new parameters have been proposed during the last decade, such as the number of involved lymph nodes [8], the number of negative lymph nodes [9], and the lymph node ratio (LNR) [10, 11]. LNR was defined as the ratio of the number of positive lymph nodes to the total number of lymph nodes examined. Several studies have proven that the LNR may serve as a better predictor of survival in patients with CRC because it is less affected by the total number of retrieved nodes [10, 12–14]. Therefore, as an alternative or complementary method, LNR have been suggested for AJCC staging [15]. It aims to improve the prognosis for CRC by reducing the effect of heterogeneity of procedures on staging lymph nodes. In addition, LNR can be a strong predictor of survival in patients with CRC, which confers additional information regarding the total number of lymph nodes examined. However, clinical node negative (cN0) patients, similar to pN0 patients, fail to benefit from the LNR system. The log odds of positive lymph nodes (LODDS) defined as the log of the ratio between the number of positive nodes and the number of negative nodes, was first proposed by Vinh-Hung V and colleagues to predict prognosis of breast cancer. In this study, it was noted that the LODDS performed equally well as a prognostic indicator in pathological lymph node status (negative [pN0] or positive [pN+]) [16]. This initial finding was subsequently extended to several kinds of cancers including CRC [17–22]. The LODDS classification was an excellent independent prognostic factor for patients with CRC, particularly those who had < 12 harvested or no lymph node metastasis [23–25]. However, some studies reported that LODDS were not related to the survival of CRC patients [26].

Considering the current controversies regarding the significance of LODDS in the prognosis of CRC patients, we systematically analyzed data obtained in published literature and summed the prognostic significance of LODDS in CRC patients.

## Materials and methods

### Study selection

We systematically searched PubMed, Medline, Embase, Web of Science and the Cochrane Library for relevant studies from inception to December 3, 2021. The following keywords were used: “log odds of positive lymph nodes”, “Colonic Neoplasms” [Mesh], and “Rectal Neoplasms” [Mesh], “Colorectal Neoplasms” [Mesh]. We used the following strategy: ((log odds of positive lymph nodes) OR (LODDS)) AND ((((((((((((“Colonic Neoplasms”[Mesh]) OR (“Rectal Neoplasms”[Mesh])) OR (“Colorectal Neoplasms”[Mesh])) OR (Rectal Neoplasms)) OR (Rectal Cancer)) OR (Rectal Tumor)) OR (Colonic Neoplasms)) OR (Colon Cancer)) OR (Colon Tumor)) OR (Colorectal Neoplasms)) OR (Colorectal Cancer)) OR (Colorectal Tumor)). For the meta-analysis, we followed PRISMA (Preferred Reporting Items for Systematic Reviews and Meta-analyses) guidelines [27].

### Inclusion and exclusion criteria

Studies fulfilling the following criteria were included: (i) the article reported at least one of the outcomes of interest or the outcome could be calculated according to data extracted from the published data; (ii) only articles published in English, focused on human, and reporting at least one outcome of interest were evaluated, or the outcome could be calculated according to data extracted from the published data; (iii) all CRC patients were diagnosed with the gold standard test; (iv) we included the studies which classified LODDS into three hierarchical levels because currently classification of LODDS has no uniform standard and we found that most of the studies classified LODDS into three categories during the study selection process.

Articles were excluded based on the following criteria: (i) missed crucial information needed for detailed stratification; (ii) number of participants less than 20; (iii) the article was a review, case report, comment, letter, or meeting record; (iv) the article shared a study population with another article.

### Data extraction and definitions

Two reviewers independently used a standardized form to extract the data from the included articles: reference, published year, country, type of cancer, number of patients (male/female), age, gender, treatment and prognostic indicators (overall survival (OS) and disease-free survival (DFS)). Any disputes or differences were settled by a third independent investigator. For articles with multiple arms, each arm was considered an independent data set.

### Outcomes and quality assessment

Prognostic values (OS and DFS) were used to compare the different LODDS groups.

Two investigators independently assessed the quality of the included articles according to the Newcastle-Ottawa scale (NOS) [28], on the basis of three categories: (i) study group selection; (ii) comparability of groups; and (iii) outcome of interest. The full score was 9, and 1–4 points indicated low-quality, while 5–9 points were considered high-quality.

### Data analysis and statistical methods

We used Stata statistical software Version 16.0 (Stata Corporation, College Station, TX) to analyze the data in our meta-analysis. To statistically assess the prognostic effects of LODDS, we extracted the hazard ratio (HR) and 95% confidence interval (CI) of OS and DFS from the included studies. If HRs, 95% CIs, or *P* values were not directly provided in the original literature, the estimated HR was used to assess prognostic effects based on the method described by Tierney et al. [29], and HR > 1 indicated more disease progression or deaths in the patients. Data were pooled using a random-effects model (REM). All statistical values were combined with 95% CIs and two-sided *P* values, the threshold of which was set to 0.05. Heterogeneity between articles was calculated using the Q test and *I*^*2*^ statistic [30]. For the *I*^*2*^ statistic, heterogeneity was defined as low (25–50%), moderate (50–75%) or high (> 75%) [31]. For the Q statistic, *P* ≤ 0.1 was considered to indicate significant heterogeneity. In addition, based on the differences in the data retrieved, subgroup analyses were performed. Then, we also conducted a sensitivity analysis in which each study was removed in turn to evaluate the undue influence of the study on the overall summary estimates including Duval and Tweedie’s trim-and-fill method [32], and Galbraith plots [33]. Publication bias was investigated with qualitative and quantitative methods, including funnel plots and Egger’s test [34]. *P* values for pooled results were two-sided, and the inspection level was 0.05.

## Results

### Study characteristics

**Fig. 1:**
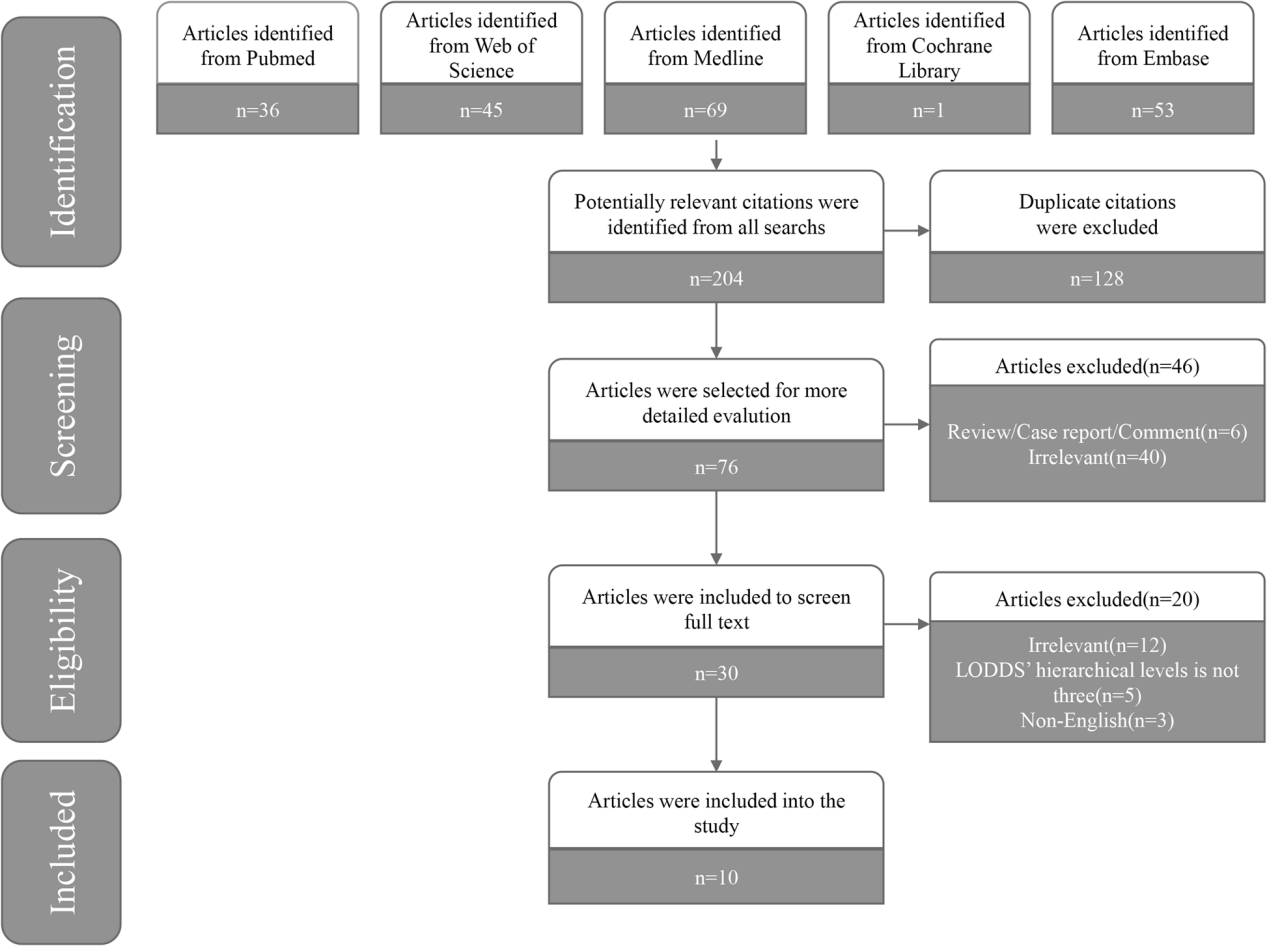
Flow diagram of study selection

The original search yielded 204 records in PubMed, Web of Science, Medline, the Cochrane Library and Embase. Of these, 128 duplicate articles were excluded. We excluded 46 records after reading the titles and abstracts. After reviewing the full texts, 10 articles [10–12, 14, 23, 24, 35–38] were finally included in this study. The flowchart of the search and selection process is demonstrated as a PRISMA flowchart in Fig. 1. All articles were published between 2012 and 2021. Overall, the 10 articles included 3523 patients, ranging from 117 to 856 patients. Among these articles, the NOS quality scores ranged from 6 to 7. The characteristics of the selected articles are detailed in Table 1.

**Table 1:** Characteristics of included studies for the meta-analyses

reference	year	single-center/multicenter	clinical study design	country	patient year	patient number		age (years)	population	follow-up (mouth)	follow-up	Cutoff	Groups in the study			NOS score
						number	male/female						LODDS0	LODDS1	LODDS2	
Arslan NC [23]	2014	single-center	prospective	Turkey	2005-2011	440	253/167	median 66 (18–96)	underwent curative resection of the colon for primary colon carcinoma	median 30.6 (0– 88)	OS	LODDS0: ≤ − 1.36 LODDS1: − 1.36 to − 0.53 LODDS2: > − 0.53	182	146	99	8
Baqar AR [35]	2020	single-center	retrospective	Australia	2011-2016	856	402/454	median 73 (22–100)	consecutive patients treated for colon adenocarcinoma	median 27.1 (0.1–71)	OS	LODDS0: ≤ − 1.36 LODDS1: − 1.36 to − 0.53 LODDS2: > − 0.53	569	217	75	8
Fang HY [36]	2017	single-center	retrospective	China	2007-2010	192	113/79	median 59 (23-90)	CRC patients who underwent curative (R0) resection	median 65 (4-106)	OS	LODDS0: ≤ − 0.82 LODDS1: − 0.82 to − 0.57 LODDS2: > − 0.57	120	17	55	8
Fortea-Sanchis C [11]	2018	multicenter	retrospective	Spain	2004-2007	548	296/252	median 72 (63–80)	diagnosed with colon cancer, undergoing surgery with curative intent, and had a complete anatomopathological report	median 51 (30–64)	OS, DFS	LODDS0: ≤ − 2 LODDS1: − 2 to 1 LODDS2: > 1	349	187	12	8
Lee CW [37]	2016	single-center	Retrospective	American	1995-2013	164	97/67	median 55 (25–95)	stage III rectal cancer patients who underwent curative resection	NR	OS	LODDS0: ≤ − 1.2788 LODDS1: − 1.2788 to − 0.7105 LODDS2: > − 0.7105	73	43	48	8
Persiani R [24]	2012	single-center	Retrospective	Italy	2004-2008	236	98/138	NC	colon cancer patients who had undergone surgical resection	median 26 (2–76)	OS	LODDS0: ≤ − 1.36 LODDS1: − 1.36 to − 0.53 LODDS2: > − 0.53	93	79	42	6
Occhionorelli S [10]	2018	single-center	Retrospective	Italy	2003-2013	202	98/104	median 76	underwent urgent colonic resection for complicated colon cancer	mean 64 (1–154)	OS, DFS	LODDS0: ≤ − 1.36 LODDS1: − 1.36 to − 0.53 LODDS2: > − 0.53	89	80	33	8
Ogawa T [38]	2015	single-center	Retrospective	Japan	1998-2011	117	54/63	Mean ± SD 61 ± 11	Stage IV CRC patients who underwent curative resection	median 51 (4–185)	OS, DFS	LODDS0: ≤ −1.133 LODDS1: − 1.133 to −0.649 LODDS2: > − 0.649	39	39	39	8
Scarinci A [14]	2018	single-center	Retrospective	Italy	2010-2015	323	172/151	Mean ± SD 72 ± 11.2	patients with primary colon or rectal adenocarcinoma that underwent curative resection	median 38 (6–67)	OS	LODDS0: ≤ −1.36 LODDS1: − 1.36 to −0.53 LODDS2: > − 0.53	165	85	73	8
Xu T [References]	2021	single-center	Retrospective	China	2004-2015	445	294/151	median 55 (23–81)	patients with locally advanced rectal cancer who received Neoadjuvant chemoradiotherapy and underwent radical surgery	median 46.7 (12.2– 148.7)	DFS	LODDS0: ≤ −1.1 LODDS1: − 1.1 to −0.6 LODDS2: > − 0.6	291	102	52	8

*Abbreviations*: *CRC* Colorectal cancer, *HR* Hazard ratio, *OS* Overall survival, *DFS* Disease-free survival, *LODDS* Log odds of positive lymph nodes

### Study analysis

We analyzed OS and DFS in different LODDS categories according to the data from the included articles [10–12, 14, 23, 24, 35–38]. The results of the pooled analysis are summarized in Table 2.

**Table 2:** Results of prognostic effects of CRC patients

Group	Studies(N)	*I*_*2*_ statistic	Model Selected	HR [95% CI]	Egger’s Test *p* Value
OS					
LODDS1 versus LODDS0	9	18.30%	Random	1.77 (1.38, 2.28)	0.729
LODDS2 versus LODDS0	9	0.00%	Random	3.49 (2.88, 4.23)	0.265
DFS					
LODDS1 versus LODDS0	4	35.00%	Random	1.82 (1.23, 2.68)	0.860
LODDS2 versus LODDS0	3	0.00%	Random	4.53 (3.14, 6.55)	0.949

*Abbreviations*: *CRC* Colorectal cancer, *HR* Hazard ratio, *OS* Overall survival, *DFS* Disease-free survival, *LODDS* Log odds of positive lymph nodes

### OS based on LODDS comparing LODDS0 versus LODDS1 and LODDS2 group

**Fig. 2:**
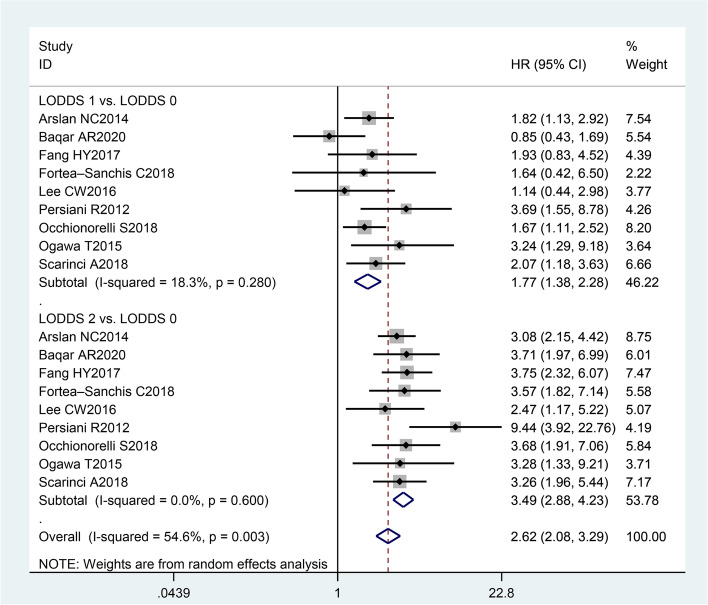
Estimated HR summary for OS. Data were pooled using a random-effects model (REM). All statistical values were combined with 95% CIs and two-sided P-values, the threshold of which was set to 0.05. HR, hazard ratio; OS, overall survival; LODDS, log odds of positive lymph nodes

Compared with LOODS0 CRC patients, LODDS1 CRC patients had a worse OS (HR = 1.77, 95% CI (1.38, 2.28)) where the heterogeneity was insignificant (*I*^*2*^ statistic = 18.3%, *P*_*heterogeneity*_ = 0.280). The pooled results indicated that LODDS2 CRC patients had a worse OS (HR = 3.49, 95% CI (2.88, 4.23)) than LOODS0 CRC patients. Regarding the heterogeneity, there was no statistical significance (*I*^*2*^ statistic = 0.0%, *P*_*heterogeneity*_ = 0.600), as shown in Fig. 2.

### DFS based on LODDS comparing LODDS0 versus LODDS1 and LODDS2 group

**Fig. 3:**
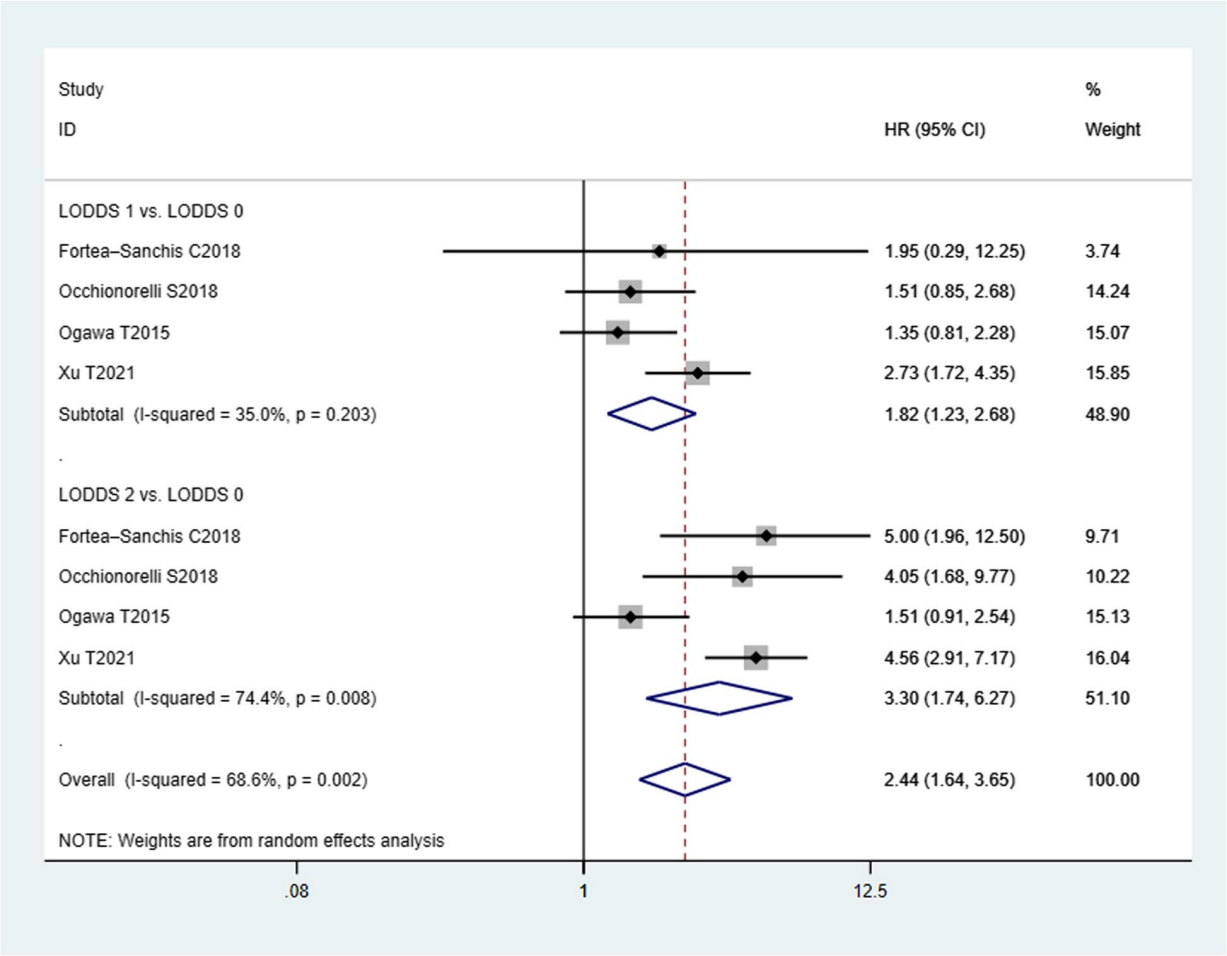
Estimated HR summary for DFS. Data were pooled using a random-effects model (REM). All statistical values were combined with 95% CIs and two-sided *P*-values, the threshold of which was set to 0.05. HR, hazard ratio; DFS, disease-free survival; LODDS, log odds of positive lymph nodes

Compared with LOODS0 CRC patients, LODDS1 CRC patients had a worse DFS (HR = 1.82, 95% CI (1.23, 2.68)). The heterogeneity was moderate insignificant (*I*^*2*^ statistic = 35.0%, P _*heterogeneity*_ = 0.203). The result of pooled analysis using the random-effects model showed that LODDS2 CRC patients was also associated with poor DFS (HR =3.30, 95% CI (1.74, 6.27)) than LODDS0 CRC patients, and between-study heterogeneity was obvious (*I*^*2*^ statistic = 74.4%, P _*heterogeneity*_ = 0.002), as shown in Fig. 3.

### The source of heterogeneity

**Fig. 4:**
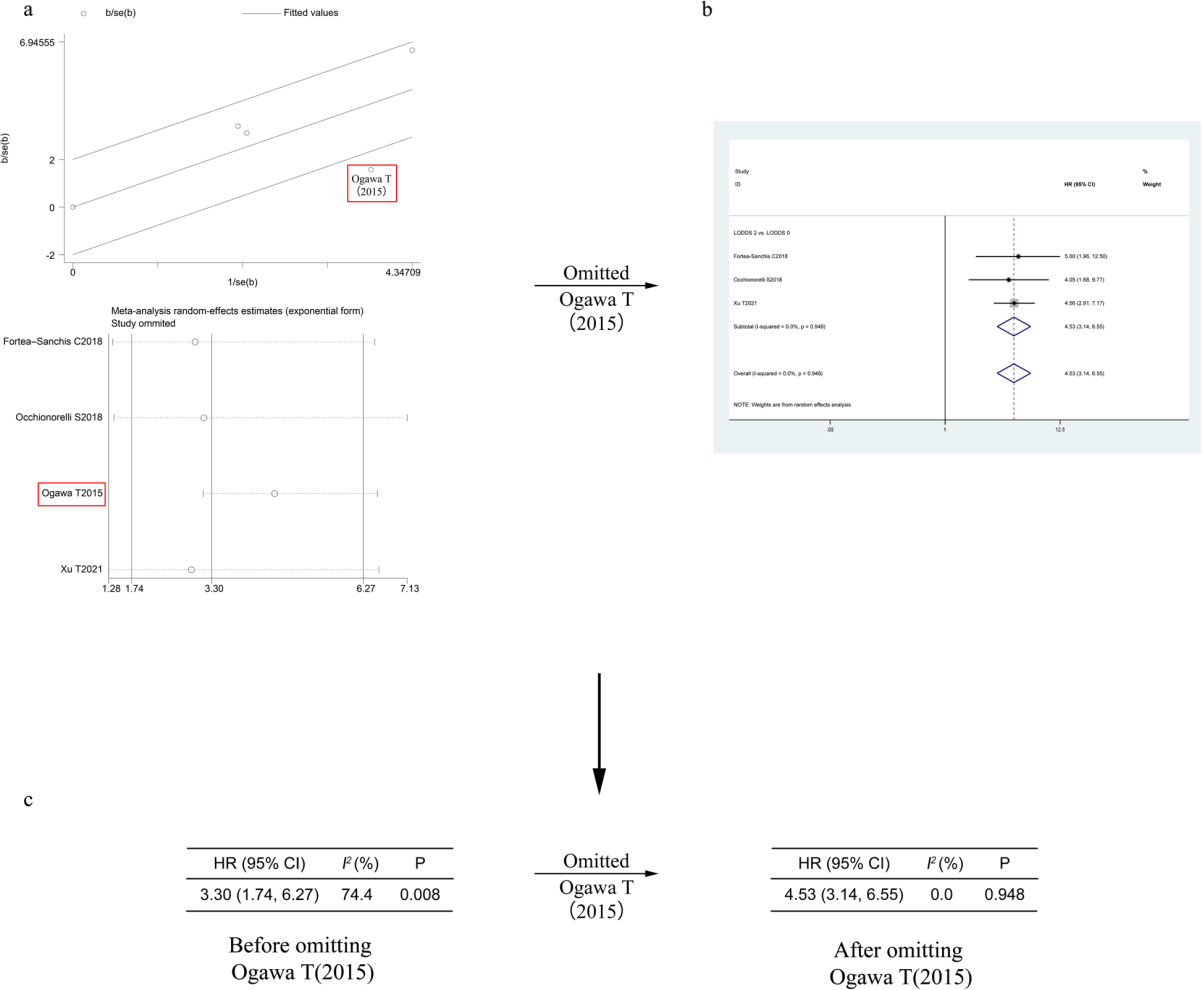
Process of exploring the potential sources of heterogeneity on DFS. **a** galbraith plot for DFS. **b** Forest plot for DFS after Ogawa S et al. (2015) is omitted. **c** change of heterogeneity before and after Ogawa S et al. (2015) is omitted. Weights are from random-effects analysis. *P* value for heterogeneity. HR, hazard ratio; DFS, disease-free survival; SE, standard error

To explore the potential sources of heterogeneity, we used Galbraith plot and Duval and Tweedie’s trim-and-fill method to further explore the source of heterogeneity in DFS, and the result showed that the training set of the study by Ogawa T et al. [38] might have mainly contributed substantial heterogeneity to DFS (Fig. 4A). After omitting this study, the pooled HR was not affected obviously (HR =4.53, 95% CI (3.14, 6.55); Fig. 4B), but the heterogeneity for DFS dropped to an insignificant level (from *I*^*2*^ statistic = 74.4%, P _*heterogeneity*_ = 0.002 to *I*^*2*^ statistic = 0.0%, P _*heterogeneity*_ = 0.948; Fig. 4C).

### Subgroup analysis and publication bias

We performed subgroup analysis according to differences in the variables, including the publication year, country, and type of cancer. Consistent with above results, LODDS1 and LODDS2 CRC patients had a worse OS and DFS compared with LODDS0 CRC patients in most subsets. Although it is found that OS and DFS of non-Asian CRC patients were better than patients from Asian, high LODDS is a marker for poor prognosis both in non-Asian and Asian CRC patients. Meanwhile, although OS and DFS of rectal cancer patients were better than colon cancer patients, high LODDS is a marker for poor prognosis both in colon and rectal cancer patients, as shown in Table 3.

**Table 3:** Results of subgroup analyses on prognostic effects of CRC patients

Comparisons (vs LODDS0)	OS			DFS		
	No. of studies	HR (95% CI)	Heterogeneity	No. of studies	HR (95% CI)	Heterogeneity
			I^2^ (%)			I^2 (%)^
**TOTAL studies**						
LODDS1	9	1.77 (1.38, 2.28)	18.3	4	1.82 (1.23, 2.68)	35
LODDS2	9	3.49 (2.88, 4.23)	0	3	4.53 (3.14, 6.55)	0
**Year**^**c**^						
> Median						
LODDS1	4	1.53 (1.07, 2.21)	25.9	1	2.73 (1.72, 4.34)	–
LODDS2	4	3.51 (2.59, 4.76)	0	1	4.56 (2.91, 7.16)	–
≤ Median						
LODDS1	5	2.06 (1.46, 2.92)	7.1	3	1.44 (0.99, 2.09)	0
LODDS2	5	3.62 (2.56, 5.11)	36.4	2	4.48 (2.36, 8.47)	0
**Country**						
East Asia						
LODDS1	2	2.41 (1.27, 4.57)	0	2	1.94 (0.97, 3.86)	74.6
LODDS2	2	3.65 (2.37, 5.62)	0	1	4.56 (2.91, 7.16)	–
non-East Asia						
LODDS1	7	1.69 (1.27, 4.57)	26.4	2	1.54 (0.89, 2.67)	0
LODDS2	7	3.47 (2.78, 4.34)	4.9	2	4.48 (2.36, 8.47)	0
**Type of cancer**						
colorectal cancer						
LODDS1	3	2.21 (1.45, 3.37)	0	1	1.54 (0.89, 2.67)	–
LODDS2	3	3.48 (2.51, 4.84)	0	1	4.56 (2.91, 7.16)	–
colon cancer						
LODDS1	5	1.68 (1.14, 2.46)	42.8	2	2.73 (1.72, 4.34)	0
LODDS2	5	3.79 (2.79, 5.15)	31	2	4.48 (2.36, 8.47)	0
rectal cancer						
LODDS1	1	1.14 (0.44, 2.98)	–	1	1.35 (0.80, 2.26)	–
LODDS2	1	2.47 (1.17, 5.22)	–	–	–	–

*Abbreviations*: *CRC* Colorectal cancer, *HR* Hazard ratio, *OS* Overall survival, *DFS* Disease-free survival, *LODDS* Log odds of positive lymph nodes

“-”: not available

^a^*P*-value for estimates of HR

^b^*P*-value for heterogeneity

^c^The median year of OS, and DFS was 2017, and 2018, respectively

**Fig. 5:**
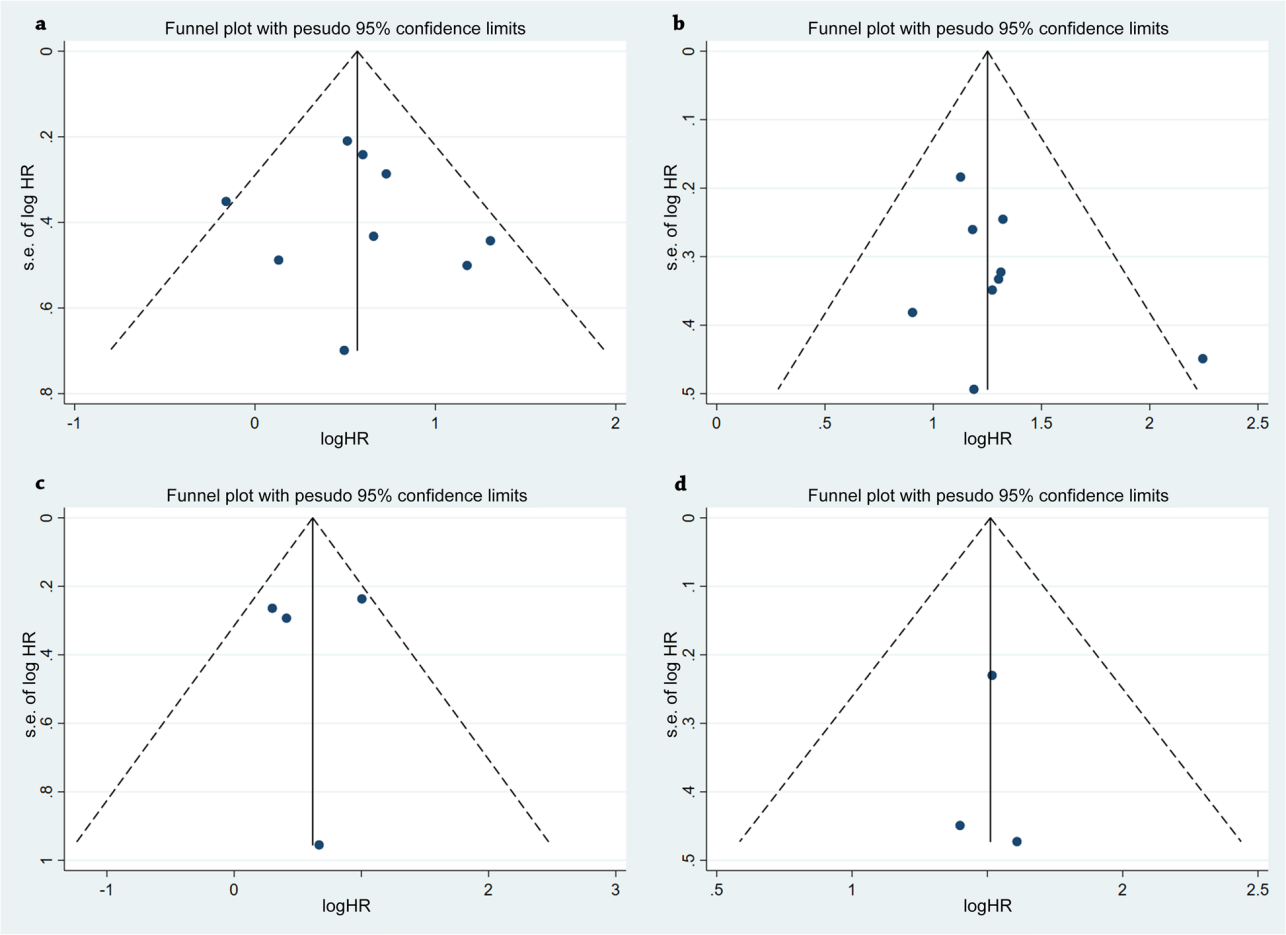
Assessment of publication bias using Funnel plot analysis. **a-b** Funnel plot analysis of studies on OS ((**a**) LODDS1 vs. LODDS0, **b** LODDS2 vs. LODDS0). **c-d** Funnel plot analysis of studies on DFS ((**c**) LODDS1 vs. LODDS0, **d** LODDS2 vs. LODDS0). Publication bias was not found in the meta-analyses of OS and DFS. All of the funnel plots of the included articles showed a symmetrical distribution. Thus, no significant publication bias was found in the meta-analyses of OS or DFS. HR, hazard ratio; OS, overall survival; DFS, disease-free survival; LODDS, log odds of positive lymph nodes

Publication bias was assessed by funnel plots and Egger’ s test, as shown in Fig. S1. Formal evaluation using Egger’ s test also failed to identify significant publication bias in the analysis of LODDS1 versus LODDS0 (*p* = 0.729), LODDS2 versus LODDS0 (*p* = 0.265) in OS. Similarly, there was no evidence for significant publication bias in LODDS1 versus LODDS0 (*p* = 0.860), LODDS2 versus LODDS0 (*p* = 0.949) in DFS. The results with heterogeneity adjusted are listed in Table 2. In addition, we used funnel plots to detect publication bias, as shown in Fig. 5. All of the funnel plots of the included articles showed a symmetrical distribution. Thus, no significant publication bias was found in the meta-analyses of OS or DFS.

## Discussion

To our knowledge, this is the first meta-analysis that focused on the significance of LODDS in the prognosis of CRC patients. Arslan NC [23] suggested that the LODDS classification was an excellent independent prognostic factor for patients with CRC, particularly those who had < 12 harvested or no lymph node metastasis. However, Jung W [26] indicated that LODDS were not related to the survival of CRC patients. Our meta-analysis of 10 articles including 3523 patients with CRC indicating that LODDS1 and LODDS2 patients had a worse OS and DFS compared with LODDS0 patients, which showed that LODDS is associated with the prognosis of CRC patients and accurately predicts survival of CRC patients. Compared with LOODS0 CRC patients, LODDS1 (HR = 1.77, 95% CI (1.38, 2.28)) and LODDS2 (HR = 3.49, 95% CI (2.88, 4.23)) CRC patients had a worse OS with insignificant heterogeneity. Additionally, the pooled results demonstrated that LODDS1 CRC patients had a worse DFS (HR = 1.82, 95% CI (1.23, 2.68)) than LOODS0 CRC patients where the heterogeneity was insignificant. Our pooled analysis also showed that LODDS2 CRC patients was also associated with poor DFS (HR =3.30, 95% CI (1.74, 6.27)) than LODDS0 CRC patients, and between-study heterogeneity was obvious (*I*^*2*^ statistic = 74.4%, P _*heterogeneity*_ = 0.002). To explore the potential sources of heterogeneity, we used Galbraith plot and Duval and Tweedie’s trim-and-fill method to further explore the source of heterogeneity in DFS, and the result showed that the training set of the study by Ogawa T et al. [38] might have mainly contributed substantial heterogeneity to DFS. After omitting this study, the heterogeneity for DFS dropped to an insignificant level (from *I*^*2*^ statistic = 74.4%, P _*heterogeneity*_ = 0.002 to *I*^*2*^ statistic = 0.0%, P _*heterogeneity*_ = 0.948; Fig. 4C), and the pooled HR was not affected obviously (HR =4.53, 95% CI (3.14, 6.55); Fig. 4B). Most results of the subgroup analysis in our study were in agreement with the survival results described above.

Despite recent advances in novel antitumor therapeutics, the overall survival is far from satisfactory, especially in patients with advanced CRC. To improve the quality of life of oncological patients, it is necessary to accurately estimate prognosis and adopt personalized therapeutics. Although the number-based UICC/AJCC pN classification in patients with radically resected CRC is currently considered as the most reliable predictor of poor prognosis [2, 3], the primary flaw of the pN classification is that the accuracy of the predicting prognosis was significantly influenced by the total number of nodes retrieved [5–7]. Neither the LNR nor pN classification system provided additional prognostic information for patients with N0 status or harvested total lymph nodes (TLNs) < 12. Recently, an increasing number of studies have confirmed the crucial roles of LODDS in the management of several types of cancer, including CRC [39–44]. LODDS, first proposed in breast cancer in which it performed equally well as a prognostic indicator in node-positive and node-negative patients [16], was later generalized to several cancers, including CRC [17–22]. The LODDS classification was a novel prognostic LN-related index that considers the effects of both the numbers of positive LNs and negative LNs and gives a new chance to improve the accuracy of pN classification for prognostic assessment, particularly in patients with N0 status or harvested < 12 TLNs [45]. By searching the most recent articles considering the prognostic value of LODDS, we found that LODDS is superior to other lymph node–based staging algorithms in predicting prognosis in several cancers. For instance, LODDS demonstrated the highest discriminative capacity and prognostic accuracy for esophageal squamous cell carcinoma (ESCC) patients [46]. Another recent study showed that LODDS was also an independent and superior predictor for OS in head and neck cancer (HNC) in a population-based setting with representative real-life data [47]. However, some studies reported that LODDS were not related to the survival of CRC patients [26]. However, several reasons may be partly explained the inconsistent conclusions of different studies, such as methodological reasons and confounder variables. In view of this, synthesizing all related findings to draw more reliable conclusions would be of interest. To our knowledge, no meta-analysis has examined the significance of LODDS in the prognosis of CRC patients. Therefore, our meta-analysis was the first and most full-scale systematic review and meta-analysis to evaluate the prognostic value of the LODDS in patients with CRC.

However, several limitations of the current meta-analysis should be emphasized. First, because several studies did not report HRs that were estimated based on the method described by Tierney et al. [29]. Second, the optimal cutoff point of LODDS need to be confirmed in a large-scale, international, multicenter prospective study before its promotion for clinical practice. Third, there were an insufficient number of studies to assess the 5-year survival rates of patients with different pN and ratio-based lymph node system (rN) classifications stratified by LODDS. That is, we were not able to access differences in survival among patients in different LODDS classification for patients in each of the pN or rN classifications. Despite these limitations, this is the first meta-analysis of focusing on the crucial roles of LODDS in predicting prognosis of patients with CRC. It is clear that LODDS accurately predicts survival of CRC patients. Moreover, it may be novel prognostic predictor, as a more accurate and sensitive stratification tool for use in clinical studies and in evaluating the appropriateness of chemotherapy treatment in homogenous patient groups.

## Conclusions

In conclusion, our systematic review demonstrated that LODDS is associated with the prognosis of CRC patients and accurately predicts survival of CRC patients. Our meta-analysis indicated that LODDS1 and LODDS2 patients have a poorer OS and DFS compared with LODDS0 patients. Moreover, the results of summary analysis demonstrated the significance of LODDS as a remarkable prognostic indicator of OS and DFS in most subgroups. Further high-quality, large-scale, international, well-designed multicenter prospective studies are required to obtain the optimal cutoff point of LODDS until the utilization of LODSS in the clinical practice.

## Supplementary Information

**Additional file 1: Figure S1.** Egger’s funnel plots on OS and DFS. a-b Egger’s Funnel plot analysis of studies on OS ((a) LODDS1 vs. LODDS0, (b) LODDS2 vs. LODDS0). c-d Egger’s funnel plot analysis of studies on DFS ((c) LODDS1 vs. LODDS0, (d) LODDS2 vs. LODDS0). HR, hazard ratio; OS, overall survival; DFS, disease-free survival; LODDS, log odds of positive lymph nodes; SE, standard error; SND, standard normal deviate.

## Data Availability

All data generated or analyzed during this study are included in this published article [and its supplementary information files].

## Abbreviations

CRC: Colorectal cancer
LODDS: Log odds of positive lymph nodes
CI: Confidence interval
OS: Overall survival
DFS: Disease-free survival
UICC: Union for International Cancer Control
AJCC: American Joint Committee on Cancer
LN: Lymph node
LNR: Lymph node ratio
NOS: Newcastle-Ottawa scale
REM: Random-effects model
HR: Hazard ratio
ESCC: Esophageal squamous cell carcinoma
HNC: Head and neck cancer
GC: Gastric cancer
pN: Pathological N
rN: Ratio-based lymph node
