# Supplementary material for: Log odds of positive lymph nodes as a novel prognostic predictor for colorectal cancer: a systematic review and meta-analysis
Source: BMC Cancer. 2022 Mar 18;22:290. doi: 10.1186/s12885-022-09390-x (PMC8932253; doi:10.1186/s12885-022-09390-x)
Supplement: Supplementary file 1 — Additional file 1: Figure S1. Egger’s funnel plots on OS and DFS. a-b Egger’s Funnel plot analysis of studies on OS ((a) LODDS1 vs. LODDS0, (b) LODDS2 vs. LODDS0). c-d Egger’s funnel plot analysis of studies on DFS ((c) LODDS1 vs. LODDS0, (d) LODDS2 vs. LODDS0). HR, hazard ratio; OS, overall survival; DFS, disease-free survival; LODDS, log odds of positive lymph nodes; SE, standard error; SND, standard normal deviate. [file 12885_2022_9390_MOESM1_ESM.docx]

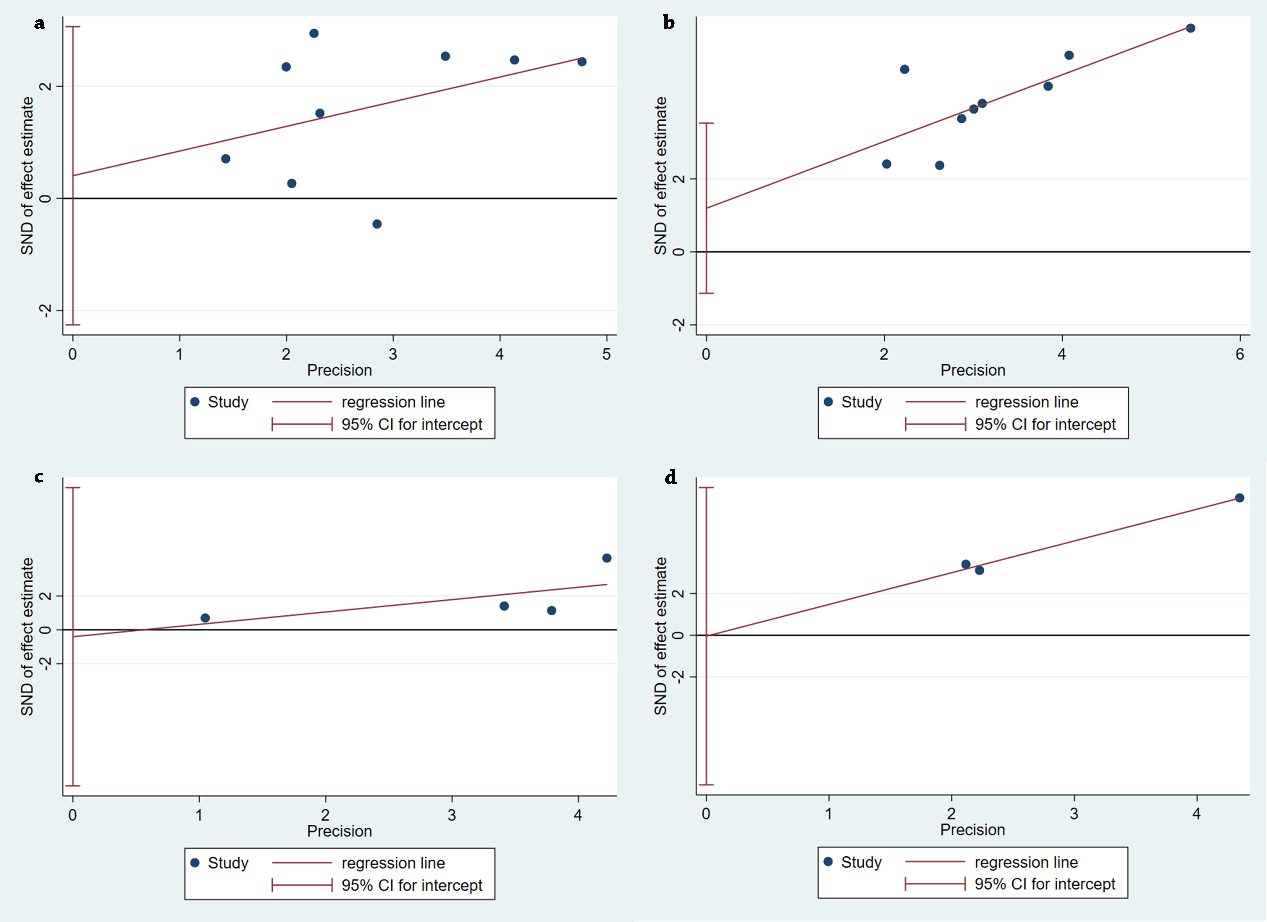


**Figure S1** Egger’s funnel plots on OS and DFS. a-b Egger’s Funnel plot analysis of studies on OS ((a) LODDS1 vs. LODDS0, (b) LODDS2 vs. LODDS0). c-d Egger’s funnel plot analysis of studies on DFS ((c) LODDS1 vs. LODDS0, (d) LODDS2 vs. LODDS0). HR, hazard ratio; OS, overall survival; DFS, disease-free survival; LODDS, log odds of positive lymph nodes; SE, standard error; SND, standard normal deviate.
